# Ricinus communis biocompatibility histological study in the nose of *Cebus apella* monkeys

**DOI:** 10.1016/S1808-8694(15)30650-9

**Published:** 2015-10-19

**Authors:** Paulo Cesar de Jesus Dias, Lidio Granato, Lizeti de Toledo de Oliveira Ramalho, José Américo de Oliveira, Hermes Pretel

**Affiliations:** 1 MSc in Otorhinolaryngology and Head and Neck Surgery - Federal University of São Paulo – Fellow in Maxillofacial Surgery - Hospital de Clinicas de Porto Alegre - Universidade Federal do Rio Grande do Sul. Phd student - Faculdade de Ciências Médicas da Santa Casa de São Paulo; 2 PhD. Professor – Otorhinolaryngology - Faculdade de Ciências Médicas da Santa Casa de São Paulo; 3 PhD. Professor – Department of Morphology - Faculdade de Odontologia de Araraquara - UNESP; 4 PhD. Professor - Núcleo de Procriação de Macacos -Prego do Departamento de Ciências Básicas da Faculdade de Odontologia do Campus de Araçatuba - UNESP; 5 MsC in Dentistry - Faculdade de Odontologia de Araraquara - UNESP – PhD student - Faculdade de Odontologia de Araraquara - UNESP

**Keywords:** castor bean, biocompatible materials, nose, polymers

## Abstract

Bone tissue lesions can be caused by congenital and acquired factors, and result in nasal deformities with cosmetic and functional repercussion. Surgical treatment in these cases frequently requires complex reconstructions and the use of biomaterials. The polyurethane derived from castor beans (Ricinus communis) has a favorable formulation in terms of ease of processing, flexibility, no emission of toxic vapors and low cost. Nonetheless, despite favorable results, studies about the use of castor beam polymer (Ricinus communis) assessing tissue reaction on the nasal dorsum are still missing in the literature.

**Aim:**

the goal of the present investigation is to histologically assess the Ricinus communis polymer implant biocompatibility with the nasal dorsum.

**Study design:**

experimental.

**Materials and Methods:**

we used four Cebus appela monkeys, in which we created a nasal dorsal defect in all the animals and there we placed the aforementioned implant. The animals were sacrificed 270 days after surgery and the samples were submitted to histological study.

**Results:**

in the histology analysis we did not observe the presence of foreign body granulomas or phagocytic cells. We also observed a progressive bone formation and maturation.

**Conclusion:**

macroscopic and microscopic results showed that the castor oil polymer implant was biocompatible.

## INTRODUCTION

Bone tissue lesions can be caused by trauma, infection, neoplasia or some congenital deformity. Craniummaxillofacial traumas are responsible for a relevant number of urgent and emergency care situations, and in some cases the patient may develop cosmetic and functional problems, needing surgery and biomaterial implants in some instances.

For quite some time the use of these material happened in an almost empirical way, and it was expected only that the material were biologically inert, with adequate mechanical resistance for the organ recovery or reconstruction.

The important biological aspects associated with autogenous grafts were discovered in the second half of the 20th century, for example, the bone morphogenetic protein^1^.

Autogenous grafts require a donor area, thus increasing morbidity and treatment risk, and it is not always possible to obtain enough volume to fill the existing bone defect, which requires additional graft placement^2^.

Biomaterial are substances or a combination of two or more pharmacologically inert natural or synthetic substances, which are used to partially or totally improve, enhance or replace tissues and organs^3^.

Among the many existing biomaterials, the castor oil-derived polyurethane has a molecular formula with aspects that favor processing; formulation flexibility; no toxic vapor emissions; good adherence power; it does not release toxic radicals when implanted, and bears low cost^4^.

Ohara et al.^5^ did not observe toxic reactions on the kidneys, liver or spleen after implanting the castor-oil-based polymer in rabbits' bones and joints.

Vilarinho et al.^6^ published that the castor bean-derived polyurethane resin was well tolerated when implanted in the anterior chamber of a mouse eye, triggering initial inflammatory reactions that reduced with time.

Figueiredo et al.^7^ carried out a comparative study between devitalized bovine bone, coral hydroxyapatite, castor bean polymer and autogenous bone grafts to fill bony defects in the femur of rabbits. The authors concluded that the castor bean polymer showed bone conductivity properties and less inflammatory reactions than devitalized bovine bone, with pore invasion and filling up of cystic cavities by bone tissue, proving to be efficient as a framework for guided tissue repair.

Porter^8^ considers the cartilaginous autograft as the standard material for nasal grafting. The following reasons were listed: the low rejection, infection or extrusion rates; ease of handling and harvesting, and the considerable quantity that can be obtained from the nasal septum or the ear pinna.

Recently, different types of materials have been used in rhinoplasty, such as polytetrafluoroethylene; Gore-Tex (the most common); silicone such as Silastic; polyethylene such as Medpore and Plastipore; and polyester such as Dacron, Mersilene and Supramid^8^, ^9^, ^10^.

According to Rodrigues^11^, the castor bean (Ricinus comunis) is a plant known in Brazil and abroad by the following synonyms: palma Christi, carrapateira, rícino, tartago. It has probably originated from India and is typical of a tropical climate. Castor beans have an important chemical oil potential, and the oil obtained from its seeds has a medicinal and cosmetic application in many fields of human need, especially biofuel. The polyurethane is derived from the polyester polyol, obtained from the vegetal fatty acid taken from the castor bean, the diphenylmethano-diisocianate.

Costa, Schall^12^ assessed the use of this polymer in urologic prosthesis in 27 patients for nine months, patients submitted to bilateral orchiectomy because of prostate cancer. The authors observed that there was no infection, foreign-body-type reaction, prosthesis breakage or extrusion. They concluded that the fibrotic capsule progression can take more than one year to evolve, therefore, more than one year is necessary in order to have a more accurate assessment; and a larger series in order to have a more satisfactory statistical study of the complications.

Suguimoto^13^ assessed the bone tissue reaction to the presence of the castor bean polymer implant in monkeys (Cebus apella). In the experiment, they used eight monkeys which received pre-conceived implants on the chin, autoclaved and fixed with titanium screws. By means of a histological analysis, the authors concluded that the castor bean polymer was biocompatible and did not induce bone neoformation in the margins or in the inside.

Puricelli et al.^14^ analyzed the castor-bean polymer behavior when implanted on the subperiosteal bed in the mandible angle of rats. The authors observed implant stability, no significant inflammatory reaction, fibrous capsule formation around the implant and bone neoformation.

Garcia Junior^15^ presented a comparative study between porous polyethylene implant (Medpore), castor bean polymer and bovine bone matrix. The experiment was carried out in eight monkeys (Cebus apella) and was aimed at assessing the bone repair process in surgical defects on the anterior wall of the maxillary sinus. 145 days after implanting, by means of histological and histometric analyses, they observed that the best histomorphological behavior was that from the bovine bone matrix, followed by the high density porous polyethylene (Medpore) and the castor being polymer (Ricinus communis), which responded with intense fibrosis and moderate late chronic inflammatory infiltrate.

Souza et al.^16^ evaluated the results from the castor bean polymer used in the reconstruction of large bone defects remaining from the resection of aggressive benign and malignant tumors in 20 patients, and after a minimum follow up of 26 and maximum of 147 weeks, they obtained 7 (35%) excellent results, four (20%) good results, four (20%) regular results and 5 (25%) failures. The authors noticed a bone neoformation in some patients, visible only after three months of postoperative.

The castor bean polymer was considered a viable alternative in this study, having the following advantages: availability, low cost, biocompatibility and osteo integration.

Cavalieri^17^ studied tissue response in bone defects created in the tibias of rabbits after implanting castor bean polymer, cement and acrylic composite. The author concluded that all three materials were well tolerated by the host tissues and reported that the castor bean polymer allowed the growth of osteogenic connective tissue inside the pores and slots, it was also incorporated to the bone tissue, bringing stability to the implant site.

Jacques18 carried out a comparative study between autogenous bone graft and castor bean polyurethane graft in rabbits submitted to a femoral-condylar pattern defect, with autogenous bone graft in of the sides and castor bean polymer in the other, randomly. In the macroscopic analysis, there was cortical bone healing in 100% of the autogenous grafts, while the group that received the castor bean polymer implant did not show this healing. Histology showed a mature bone tissue in the transition zone between the implant and the receiving bone in all the animals that received autogenous bone graft and in none of the ones that received castor bean polyurethane implants, where there was immature bone tissue. The author concluded that the castor bean polyurethane implant integrated itself to the receiving bone in a slower and more incomplete way when compared to the autogenous bone graft.

## OBJECTIVE

The present investigation aimed at histologically assessing the castor bean polymer implant biocompatibility with Cebus appella monkeys.

## MATERIALS AND METHODS

This project was previously analyzed and authorized by the Ethics in Animal Experimentation Committee, under protocol # 84. In order to perform the present investigation we used four adult Cebus paella monkeys.

The animals had a mean weight of 2kg and age between 7 and 8 years; the preparation and the anesthetic procedure were in accordance with the Institution's protocol.

During anesthesia, the animals were submitted to the inhalation of diethyl-sulphuric ether and, following that, we injected the animals with sodium thiopental intraperitoneal solution, in the concentration of 30 mg/Kg of body weight. During postoperative, the animals were kept under controlled conditions of light and moisture of 60 to 80% and room temperature - they received water and a balanced meal.

**Figure 1:**
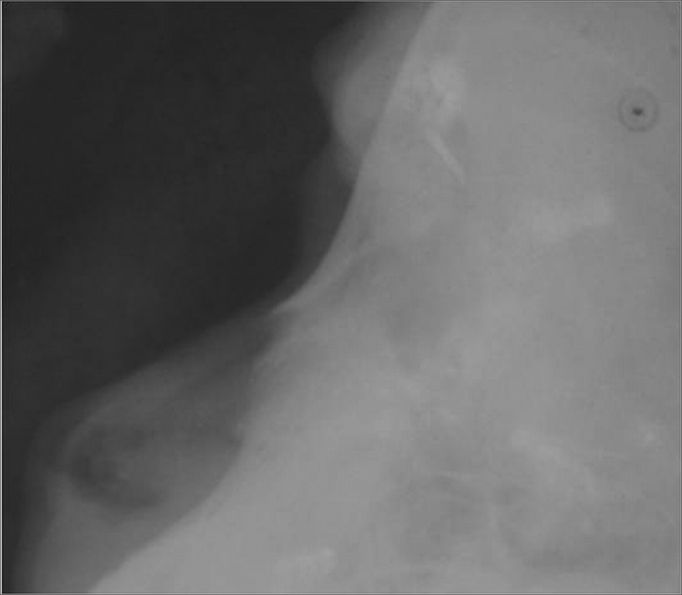
Left side lateral radiograph of the nose in the preoperative

Radiological evaluation was carried out (Spectro II DabiAtlante - Ribeirão Preto) under conventional settings of 50 KV and 10 mA, in the side-to-side position on the left profile of the face in the preoperative period (Figure 1).

**Figure 2:**
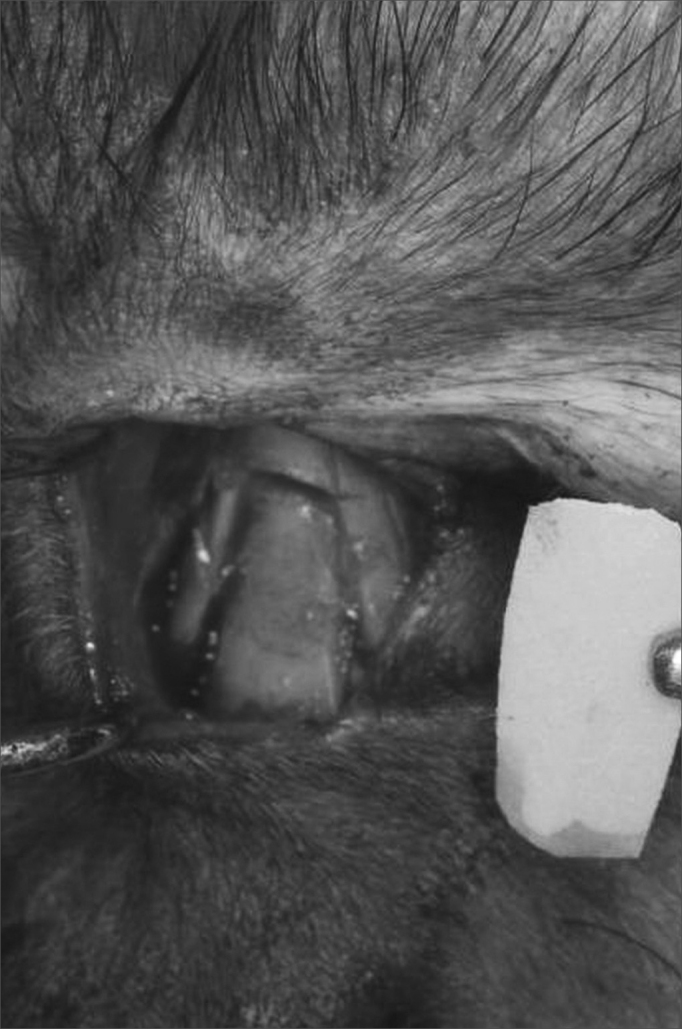
Transoperative aspect showing nasal bone fracture.

**Figure 3:**
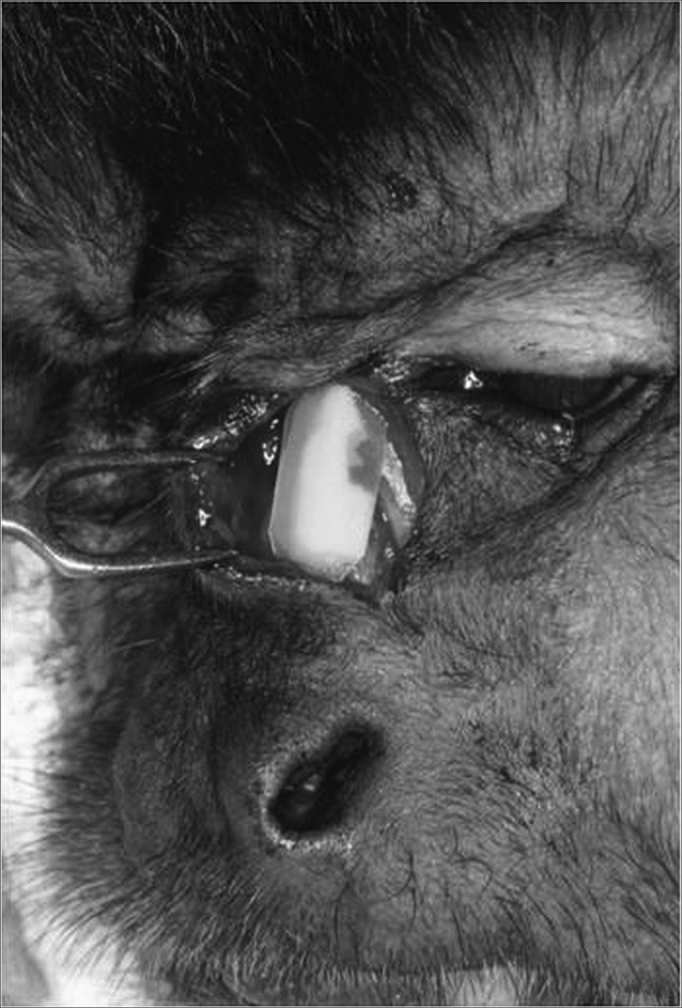
Transoperative image showing the castor bean implant on the bone defect.

**Figure 4:**
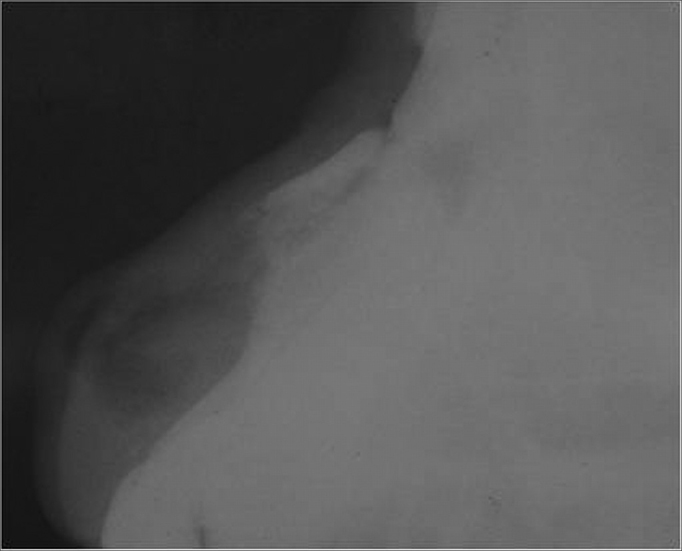
Lateral nose radiograph - immediate postoperative.

The surgical procedure followed all the antiseptic criteria used to make defects to the nasal bones - luxation (Figure 2). The castor bean polymer implant was placed on the bone defect (Figure 3) and fixed with polyglactine wire (polyvicryl 4.0). Radiologic control was performed at the end of the procedure (Figure 4). After 270 days, the animals were slaughtered and the material was referred for morphological analysis, following the routine methodology of the histology laboratory. The slides were dyed by Hematoxylin and Eosin (HE) for morphological analysis in Masson's Trichromic (MT) in order to show bone formation in response to the castor bean polymer and the Picro-sirius (PS) in order to analyze the degree of collagen maturing by polarizing microscopy.

## RESULTS

After 270 days, there was no clinical evidence of the degree or irregularity of nasal bone topography or scar retraction in the surgical wound.

**Figure 5:**
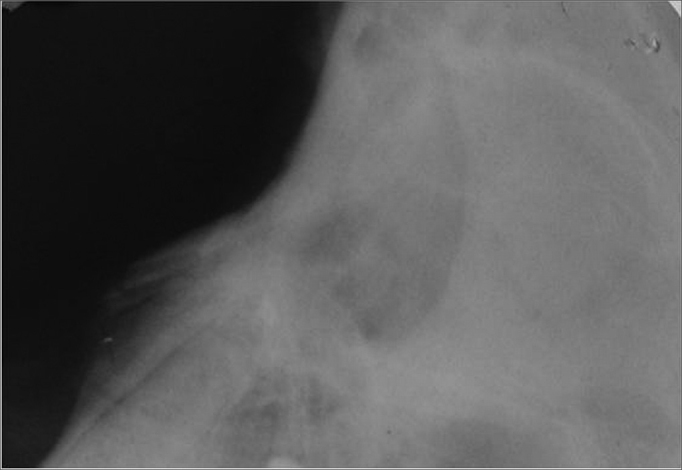
Lateral nose radiograph after 270 days.

The surgical incision scar was practically invisible in all the animals and the radiologic results on the lateral portion of the face proved nasal bone integrity (Figure 5).

**Figure 6:**
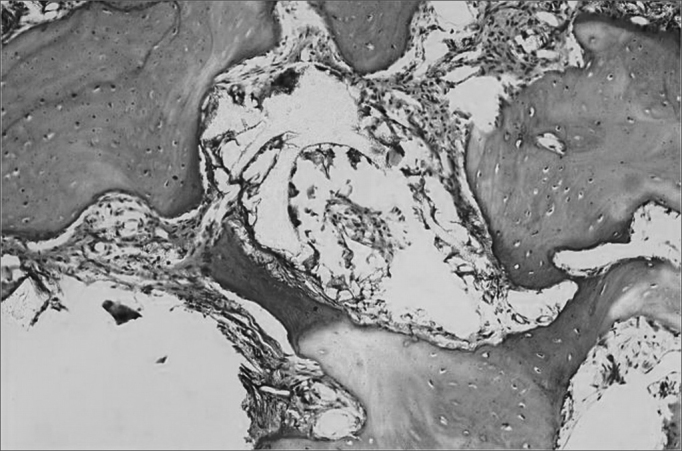
Microphotography showing castor bean polymer membrane inside the loose connective tissue. Hematoxylin-Eosin dye. 156 X magnification.

**Figure 7:**
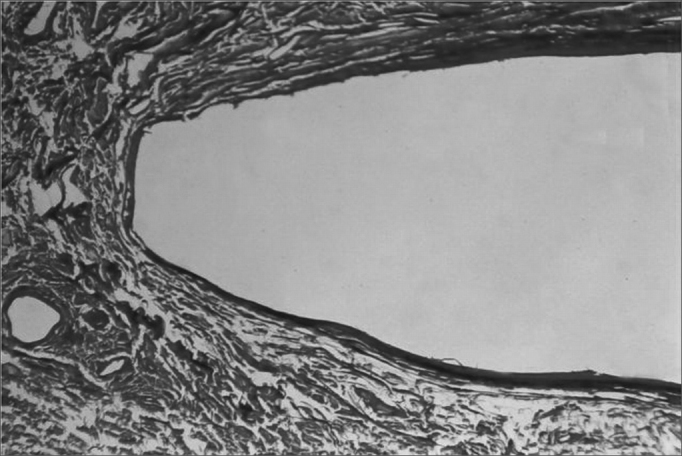
Castor bean polymer insertion site. Picro-sirius dye. 160 X magnification.

Histology of the present investigation showed that after a period of 270 days, the bone suffered a major remodeling characterized by a wealth of cells with abundant osteogenic cells. We did not see inflammatory cells or even characteristics of a foreign-body type reaction. The presence of castor bean polymer fragments amidst the neoformed bone tissue (Figure 6) and different degrees of mineralization and mature collagen fibers showed by the high degree of birefringence seen in the dye next to the castor bean polymer insertion site was not absorbed (Figure 7).

## DISCUSSION

A low cost material and abundant in nature would be ideal to replace, with advantage, the high cost material or those that require more than one surgical intervention, as in the cases of autogenous grafts. By this token, the castor bean polymer has undergone rigorous investigation as a material viable for grafts.

Studies by Vilarinho et al.^6^, Suguimoto^13^, Souza et al.^16^, Cavalieri^17^ used the castor bean polymer in the most diverse situation and animal models such as mice, rabbits, dogs and monkeys within observation periods from 10 days to 147 weeks, and there were no signs of infection or material extrusion. The literature is in agreement with the clinical findings of the present investigation, where we noticed the presence of castor bean polymer on the nasal dorsum after 270 days and it was stable and covered by bone.

Nevertheless, the fact that the implant does not suffer extrusion during the study may not correspond to the final result, since that in clinical practice there are many reports of implant extrusion from 2 months all the way to 19 years of postop^8^, ^9^, ^10^.

Costa and Shall^12^ used the castor bean polymer as urologic prosthesis and did not notice infection, foreign-body-type reactions or extrusions. These authors report that the progression to a fibrous capsule takes at least two years in order to have a more accurate assessment.

Porosity allows for tissue growth inside the material, and such fact would difficult movement and cause implant migration and extrusion^8^, ^9^, ^10^. Regarding polymer porosity, there are controversial reports; most studies report the use of the polymer associated with calcium carbonate, resulting in a porous mass when compared to the castor bean polymer membrane.

Studies by Suguimoto^13^ and those from Garcia Junior^15^ used the same animal model we used, but they used the polymer associated with calcium carbonate. In our study we used the castor bean polymer membrane. In this experiment we did not see osteogenic tissue invasion in the histology inside the implant pores. The findings disagreed from those by Cavalieri^17^; who concluded that the porosity of the implant's internal architecture allows the incorporation of the material to live tissue and favors the regeneration of bone defects.

Considering the findings in the literature, we noticed conflicting results, basically reflecting the differentiated methodological profile in each study, because of the multiple variables, such as the animal model, and the region studied in the same animal; the assessment period; the type of castor bean polymer used and the aims of the research project. Nonetheless, in most of the studies we read, we did not see implant extrusion or infection reports associated with the implant. The cases that involved one defect had bone malformation with immature bone with a trend towards the formation of collagen fibers on the castor bean polymer implant surface. We can not state that the castor bean polymer induced osteogenesis; this was probably due to the surgical trauma stimulus on the remaining bone stump and nasal bone periosteum.

## CONCLUSION

According to the methodology employed and considering the results obtained we can conclude that: the castor bean polymer was biocompatible, there was biomaterial persistence 270 days after surgery and the implant did not induce bone neoformation.
